# Executive dysfunctions in patients with low-grade gliomas in the supplementary motor area: a neuropsychological perspective

**DOI:** 10.3389/fnhum.2025.1554063

**Published:** 2025-07-18

**Authors:** Aleksandra Bala, Agnieszka Olejnik, Weronika Rejner, Antonina Gottman-Narożna, Kacper Koczyk, Tomasz Dziedzic, Przemysław Kunert

**Affiliations:** ^1^Faculty of Psychology, University of Warsaw, Warsaw, Poland; ^2^Department of Neurosurgery, Medical University of Warsaw, Warsaw, Poland; ^3^Doctoral School, Medical University of Warsaw, Warsaw, Poland

**Keywords:** supplementary motor area, SMA, low-grade glioma, LGG, executive functions, neuropsychological assessment, cognitive functions

## Abstract

**Introduction:**

The supplementary motor area (SMA) is one of the more common locations where low-grade gliomas (LGG) are found. It is an area that, in addition to controlling movement, is also involved in a range of cognitive functions, including executive functions, but data on this topic is still scarce. The aim of this study was a comprehensive assessment of executive functions, as well as an evaluation of clinical variables that may influence the obtained results.

**Materials and methods:**

The study included 23 patients with LGG tumors located in the SMA and a control group of 57 healthy individuals. They were all tested using a set of neuropsychological tests: the Stroop Test, the Verbal Fluency Test, the Tower of London Test, and the Wisconsin Card Sorting Test.

**Results:**

The conducted analyses revealed the presence of significant differences between groups in terms of selected indicators of each test. Furthermore, the results of individual TOL and WCST indicators showed significant correlations with the tumor volume. The comparison of patients with tumors in the left and right hemispheres revealed differences solely in WCST performance, with those having left SMA damage performing worse. There were also differences in the performance of the WCST test to the disadvantage of patients who had epileptic seizures compared to those who did not. Moreover, patients with oligodendrogliomas scored lower than those with astrocytomas on some of the WCST indices.

**Conclusions:**

Patients with LGG tumors in the supplementary motor area present a wide range of executive dysfunctions, including planning, reasoning, inhibition, switching, and cognitive flexibility. Both the volume and type of tumor, the hemisphere in which it is located and the occurrence of epileptic seizures may be related to the results. Future studies with larger cohorts are needed to confirm these findings.

## Introduction

Executive functions are complex cognitive processes that enable goal-directed behavior, flexible thinking, and adaptive responses to new information (Cristofori et al., 2019; Logue and Gould, 2014). Key components include, inter alia: planning, decision-making, response inhibition, reasoning and cognitive flexibility (Lezak et al., 2012; Takeuchi et al., 2013). While traditional models of executive functions emphasize the role of prefrontal cortex (Stuss, 2011), evidence increasingly highlights the involvement of other structures, including temporal lobe (Olejnik et al., 2024), cerebellum (Bellebaum and Daum, 2007), basal ganglia (Florio et al., 2018), thalamus (Van der Werf et al., 2003) and also the supplementary motor area (SMA) (Sjöberg et al., 2019).

The supplementary motor area complex, has been shown to play a crucial role not only in motor planning but also in a broad range of executive functions, including preparation, initiation, and inhibition of voluntary actions, regulation of attention and temporal processing (Bala et al., 2025; Coull et al., 2016; Duann et al., 2009; Nachev et al., 2008; Palmisciano et al., 2022). Furthermore, these regions are involved in monitoring performance and adjusting behavior in response to changing task demands, task switching, conflict monitoring, and decision-making (Aron et al., 2007; Cona and Semenza, 2017; Dominik et al., 2024; Rushworth et al., 2002; Fedota et al., 2014). Importantly, dysfunctions of the SMA have also been reported in various clinical populations beyond neurosurgical patients. For instance, altered SMA activity has been associated with attention and time estimation impairments in schizophrenia (Ortuño et al., 2005).

The SMA can be divided into two parts: SMA proper and pre-SMA, which differ in their structural and functional properties. The SMA proper, situated anterior to the primary motor cortex has extensive connections with primary motor cortex, premotor cortex and basal ganglia, making it well-suited for motor planning and execution (Lu et al., 1994; Piervincenzi et al., 2023). The pre-SMA, positioned anterior to the SMA, is distinguished by its connectivity with prefrontal and parietal cortices, basal ganglia, and cerebellum. These connections facilitate its role in higher-order motor control but also executive functions (Lu et al., 1994).

Low-Grade Gliomas (LGG) are slowly growing brain tumors that arise from glial cells, and account for ~15% of all gliomas. They are most often diagnosed among young adults, namely those aged around 20–40 years (Bauchet et al., 2024; Rimmer et al., 2024). Symptoms may include seizures, headaches, or neurological and neuropsychological deficits, depending on the tumor's location (Forst et al., 2014; Rimmer et al., 2024). Diagnosis of LGG tumors is established on the basis of a combination of neuroimaging, histopathology, and molecular diagnostic methods (Forst et al., 2014). LGGs often have a relatively good prognosis compared to high-grade gliomas (Lanese et al., 2018; Xu et al., 2022), making it especially important to prioritize the preservation of neurological and cognitive functioning. A neuropsychological examination plays a vital role in this process. It provides a comprehensive assessment of cognitive, emotional, and behavioral functioning, helping to identify subtle deficits that may impact the patient's quality of life (Ek et al., 2024; Heitzer et al., 2019). This information guides personalized treatment planning, including strategies to minimize the impact of interventions on brain function.

The supplementary motor area is a common site for low-grade gliomas (Duffau and Capelle, 2004). Data on the neuropsychological functioning of people with tumors in the SMA focus mainly on language and motor functions (Alario et al., 2006; Courson et al., 2017; Hertrich et al., 2016; Makoshi et al., 2011; Oda et al., 2021; Welniarz et al., 2019), and relatively little is known so far about the executive functioning of these patients, with existing data providing conflicting information.

Sjöberg et al. (2019) examined the relationship between the SMA syndrome and cognitive control in postoperative patients. The findings suggest that SMA symptoms are associated with marked deficits in cognitive control. Patients performing the Stroop test and verbal fluency tests exhibited significant difficulties in cognitive control both before and after surgery. The limitation of this research is that the authors only included data from five participants, which makes it impossible to generalize the conclusions to the whole population. Other researchers (Cañas et al., 2018) used verbal fluency test, also finding significant differences in the phonemic criterion but not in semantic, which is congruent with the data stating that this condition is more sensitive to the executive function deficits (Delgado-Álvarez et al., 2021). On the other hand, Tymowski et al. (2018) analyzed data from 21 patients with SMA tumors. The researchers examined the patients using a set of neuropsychological tests that covered a range of cognitive functions, including executive functions. Patients were tested before and after surgery using, among others, the Wisconsin Card Sorting Test, Ruff Figural Fluency Test, Trail Making Test and Verbal Fluency Test. No significant deficits in executive functions were found either before or after surgery in any of the tests used; the only one that showed a decline was the Verbal Fluency Test. Moreover, Guida et al. (2023) conducted a study using two non-invasive neuromodulation techniques (NIBS) to investigate the direct impact of the SMA on automatic and voluntary control. The results did not show significant differences between true and sham NIBS in both cases, suggesting that the SMA might not play a direct role in executing functioning and controlling behavior.

Taking into account the contradictory data, we decided to take a detailed look at the executive functioning of patients with LGG tumors located in SMA. It was hypothesized that patients with gliomas in this area may present a wide range of executive dysfunctions, including: impairments in cognitive control and response inhibition, tendency to perseverations, difficulties with task switching, decision-making, planning and reasoning. It was also hypothesized that the volume and lateralization of the tumor will be associated with the severity of deficits observed in evaluated patients.

## Materials and methods

### Participants

An MRI imaging revealing the tumor's location in the supplementary motor area served as the basis for initial selection to participate in the study. Initially, we included sixty-three patients whose neuroimages fit this criteria. Following surgery, the final verification was obtained by histological evaluation, and it revealed that forty patients had other types of lesions: six—meningiomas, twenty seven high grade glioma (HGG) and seven—metastatic tumors, so their data was removed from the further analyses. Finally, the study involved twenty-three patients with low grade glioma tumors (LGG) located in the supplementary motor area complex, as well as 57 healthy volunteers aged 20–80. Exclusion criteria in both cohorts—clinical group and control group were: history of any other neurological or psychiatric diseases, drug or alcohol addiction or any serious disabilities that would impair patients' ability to complete the neuropsychological assessment (e.g., major visual or hearing deficits). Medical history was assessed on the basis of the declarations of participants of the study. Current neurological and psychological status was assessed by clinical specialists during the patient's hospitalization; these data were taken into account during qualification for the study. Demographic and clinical characteristics of the participants are presented in Table 1. Statistical analyses showed that the groups did not differ in terms of any demographical variable.

**Table 1:** Demographical and clinical data.

	**Clinical group (*N =* 23)**	**Control group *(N =* 57)**	***p*-value**
Sex: M/F	12/11	30/27	> 0.05
Age M (SD)	39.78 (12.65)	41.92 (13.65)	> 0.05
Years of education M (SD)	14.08 (2.27)	14.78 (2.80)	> 0.05
Handedness: R/L	23/0	56/1	> 0.05
Tumor volume in cm^3^ M (SD)	15.33 (12.34)	N/A	N/A
Tumor lateralization: R/L	9/14	N/A	N/A
Tumor type: astrocytoma/oligodenroglioma	10/13	N/A	N/A
Epileptic seizures: yes/no	13/10	N/A	N/A

N/A, not applicable.

### Procedure

Each participant was examined individually by a trained neuropsychologist with a set of neuropsychological tests performed in the same order during a single session, 1 day prior to the surgery. Depending on each participant's physical and neurological state, the assessment time varied and took approximately from 60 to 90 min. The entire procedure was conducted in a tranquil setting within the hospital facilities during the morning hours. At the beginning of the meeting, participants were requested to provide information regarding their demographic details. The clinical data, which included information about the location and the type of the tumor, was obtained from magnetic resonance imaging (MRI) conducted during the patients' hospitalization and histopathological diagnosis obtained about two weeks after the surgery. All MRI data was analyzed manually by a trained radiologist. All procedures were carried out in accordance with the Declaration of Helsinki and were approved by the Ethics Committee of the Faculty of Psychology at the University of Warsaw (05/11/2024/15). All participants gave informed consent to participate in the study.

### Neuropsychological examination

The Victoria Stroop Test (VST; Spreen and Strauss, 1998) is a brief version of the original Stroop task (Stroop, 1935). It measures cognitive flexibility, cognitive control and the ability to inhibit an automatic response. The test consists of three parts. In each of them, a card containing six rows of four different stimuli is given and the examinee is told to do every part as fast as possible. In the first part the examinee is asked to name the color of the presented dots (red, blue, yellow, and green). In the second condition neutral common words replace the dots, but are printed in the same colors as dots in the first part. In this condition the examinee is required to name the color of the written word and omit the meaning of it. In the third part presented words are names of colors but printed in different colors than their meaning (e.g., word “green” is written in yellow). The examinee is told to name the color that the word is written in and not the meaning of it. The number of errors made are noted down and the total execution time of each part is measured.

The Wisconsin Card Sorting Test (WCST; Heaton et al., 1993) is designed to assess executive functioning, especially cognitive flexibility and reasoning. It requires strategic planning, modulating impulsive responses and the ability to shift between different possibilities depending on environmental feedback. The examinee is presented with four key cards (the first one with a red triangle, the second with two green stars, the third with three yellow crosses and the forth with four blue circles). The examinee is given two decks each containing 64 cards. Each card has a similar design that matches somehow the key cards. Cards can be distinguished by shape, color and number. The examinee is asked to place the card under one of the key cards in the way that they think the card matches the key card. Then a feedback is given by the examiner response whether the placement is correct or wrong. The examinee has to adjust their actions in order to correctly match the cards. Changes in sorting rules take place without previous warning. The examinee has to correctly place ten cards in a row for the rule to change. This procedure is administered until completion of six sorting categories or until all 128 cards are used. There is no time limit for sorting cards.

Verbal Fluency Tests (VFT; Lezak, 2004) are used to assess executive functions, lexical retrieval abilities and words production. The most common versions of verbal fluency test are phonemic fluency based on letter criterion and semantic fluency which is category guided. In phonemic verbal fluency (letter-guided) the examinee is asked to generate orally as many nouns as possible starting with a particular letter, excluding proper nouns such as people's, city and country names. In our study K letter was used. In semantic verbal fluency (category-guided) the examinee is asked to produce orally as many words from a specific category as possible. Semantic fluency tasks was conducted in two ways—using a single category (animals) or switching between two categories (vegetables and professions named alternately). The switching condition is used to evaluate cognitive flexibility, inhibition, attention and working memory. In each part, the examinee has 60 s for completing the task and is informed about the time limit.

The Tower of London test (TOL; Shallice, 1982) is designed to assess executive functions, especially planning, solving problems, working memory and response inhibition. The test consists of two boards with three pegs with different heights and three colored beads. The examinee is asked to recreate on their board the configuration of beads presented by the examiner on his board. Test includes two examples and ten tasks. The goal is to assemble a correct configuration using the minimum required moves. There are rules that need to be respected: using the examiner's board and beads is forbidden, only one movement of the bead at a time is allowed, the number of beads allowed on each peg varies depending on the height of pegs. The time of planning a first move, completing the configuration and total execution time is measured up to 120 s. The total number of movements as well as any rule violations are recorded.

### Statistical analysis

Statistical analyses were performed using the Statistical Product and Service Solutions (SPSS) version 29.02 software (IBM Corp., Armonk, NY, USA) for Windows. The Shapiro-Wilk test demonstrated that the data had a normal distribution so the parametric tests could be used as the data met also other requirements. The chi^2^ test was used to evaluate the frequency of distributions of categorical variables, and the Student's *t*-test for independent samples was used to compare the means of data collected from patients and healthy volunteers. When analyzing the correlation between continuous data, Pearson's r coefficient was employed. Results were considered significant when *p* < 0.05.

## Results

The results obtained from the comparison between patients and healthy subjects showed that patients scored lower in each of the neuropsychological tests that were used, but not in each of the analyzed indicators. Namely, significant differences were observed in the performance of the Verbal Fluency test in the phonemic and switching condition, with no significant differences in the semantic criterion. In the Stroop Test, the subjects obtained similar performance times for the particular parts of the test (performance on the third part—Stroop task showed a trend toward statistical significance, but did not reach it), but significant differences were observed for the interference score and the number of errors. In the Tower of London test, the subjects in the clinical group differed from the healthy subjects in the number of correct answers, extra moves, and errors, and did not differ in time of planning; in terms of execution time and total test completion time, the differences only indicated a trend, but did not reach statistical significance. The WSCT test showed significant differences in the number of trials, number of correct answers, perseverations and perseverative errors, number of completed categories, failure to maintain set and learning to learn indicators, while non-perseverative errors, conceptual responses and trials to complete the first category were performed at a similar level to healthy subjects. Details of the individual tests and their results can be found in Table 2.

**Table 2:** Between-group differences in cognitive testing.

**Neuropsychological tests**		**Clinical group (*N =* 23) M (SD)**	**Control group (*N =* 57) M (SD)**	**Student's *t*-value**	***p*-value**	**Cohen's *d***
Verbal Fluency	Animals	20.44 (5.67)	21.57 (3.18)	0.74	0.232	0.24
	**K letter**	**13.16 (4.04)**	**17.78 (3.64)**	**1.28**	**0.010**	**1.62**
	**Switching**	**12.70 (4.05)**	**17.00 (2.28)**	**3.67**	**0.001**	**1.43**
Stroop Test	Dots (time)	14.08 (2.23)	14.80 (2.92)	0.82	0.206	0.26
	Stroop Task (time)	29.79 (7.12)	26.64 (7.53)	−1.34	0.092^*^	−0.42
	**Interference score**	**2.09 (0.39)**	**1.81 (0.42)**	–**2.07**	**0.022**	–**0.67**
	**Errors**	**1.92 (2.12)**	**0.19 (0.70)**	–**2.98**	**0.005**	–**1.37**
Tower of London	**Correct**	**4.11 (2.58)**	**5.38 (2.55)**	**1.72**	**0.045**	**0.49**
	**Extra moves**	**31.44 (21.55)**	**22.19 (15.37)**	–**1.81**	**0.037**	–**0.52**
	Plan time	54.00 (35.21)	61.02 (38.36)	0.65	0.259	0.18
	Execution time	227.22 (126.90)	182.83 (73.06)	−1.63	0.054^*^	−0.47
	Total time	280.94 (133.16)	238.94 (69.36)	−1.53	0.066^*^	−0.44
	**Time** **>** **1min**	**1.05 (1.30)**	**0.38 (0.54)**	–**2.07**	**0.025**	–**0.76**
	**Total errors**	**0.72 (1.31)**	**0.11 (0.31)**	–**1.93**	**0.034**	–**0.76**
Wisconsin Card Sorting Test	**Number of trials**	**112.22 (24.90)**	**89.56 (27.19)**	–**2.26**	**0.014**	–**0.84**
	**Total correct**	**73.77 (11.20)**	**65.05 (16.04)**	–**1.89**	**0.038**	–**0.57**
	Total errors	38.44 (22.56)	26.82 (22.30)	−1.37	0.096^*^	−0.52
	**Perseverations**	**25.22 (17.88)**	**12.20 (9.84)**	–**2.10**	**0.032**	–**1.10**
	**Perseverative errors**	**22.44 (14.60)**	**11.65 (9.13)**	–**2.77**	**0.004**	–**1.03**
	Non-perseverative errors	16.00 (9.78)	13.06 (13.05)	−0.63	0.266	−0.23
	Conceptual responses	61.88 (14.10)	60.80 (8.80)	−0.29	0.386	−0.10
	**Completed categories**	**4.22 (1.56)**	**5.20 (1.41)**	**1.81**	**0.038**	**0.67**
	Trials to complete the first category	12.44 (3.64)	13.65 (11.35)	0.31	0.378	0.11
	**Failure to maintain set**	**1.88 (1.26)**	**0.54 (1.03)**	–**3.31**	**0.001**	–**1.23**
	**Learning to learn**	–**12.55 (17.44)**	**–3.29 (7.95)**	**2.29**	**0.014**	**0.90**

Significant results in bold;

* statistical trend; all time scores are in seconds.

Next, the relationship between tumor volume and neuropsychological test results was analyzed. It turned out that the size of the tumor was not related to the results of the Verbal Fluency test and the Stroop Test, but several significant correlations were found with selected subtests of the TOL and WCST. More specifically, in the case of TOL, significant correlations were found with the following indicators: Execution time (*r* = 0.60; *p* = 0.009), Total time (*r* = 0.66; *p* = 0.002), Time above 1 min (*r* = 0.62; *p* = 0.006), Total errors (*r* = 0.50; *p* = 0.035), and a correlation significant at the trend level with the Extra moves (*r* = 0.42; *p* = 0.07). In the case of WCST, significant correlations were detected with a number of Completed categories (*r* = −0.76; *p* = 0.017) and Learning to learn (*r* = −0.71; *p* = 0.047), as well as trend-level correlations with Total errors (*r* = 0.58; *p* = 0.09) and Non-perseverative errors (*r* = 0.59; *p* = 0.08).

The effect of tumor lateralization was analyzed. We decided to use the Student's *t*-test despite the small sample sizes, as the formal requirements such as homogeneity and normality of distribution were met. Patients with a tumor located in the left hemisphere were compared to those with a tumor in right hemisphere. Significant differences were found only in particular indicators of WCST, namely in: Total errors, Perseverations, Perseverative errors, Non-perseverative errors and Completed categories. Failure to maintain set and Learning to learn differed at the statistical trend only. Details can be found in Table 3.

**Table 3:** Comparison of the results of patients with a tumor in left and right SMA.

**Neuropsychological tests**		**Left SMA (*N =* 14) M (SD)**	**Right SMA (*N =* 9) M (SD)**	**Student's *t*-value**	***p*-value**	**Cohen's *d***
Wisconsin Card Sorting Test	Number of trials	123.00 (12.24)	90.66 (32.57)	2.25	0.111	1.59
	Total correct	74.33 (8.54)	72.66 (17.78)	0.19	0.425	0.14
	**Total errors**	**48.66 (18.73)**	**18.00 (14.79)**	**2.42**	**0.022**	**1.73**
	**Perseverations**	**32.83 (16.70)**	**10.00 (7.81)**	**2.19**	**0.032**	**1.55**
	**Perseverative errors**	**28.66 (13.32)**	**10.00 (7.81)**	**2.19**	**0.032**	**1.55**
	**Non-perseverative errors**	**20.00 (8.71)**	**8.00 (7.00)**	**2.05**	**0.040**	**1.45**
	Conceptual responses	58.00 (13.84)	69.66 (13.42)	−1.20	0.134	−0.85
	**Completed categories**	**3.50 (1.37)**	**5.66 (0.57)**	–**2.54**	**0.019**	–**1.79**
	Trials to complete the first category	11.33 (0.81)	14.66 (6.35)	−1.36	0.108	−0.96
	Failure to maintain set	2.33 (0.81)	1.00 (1.73)	1.63	0.073^*^	1.15
	Learning to learn	−20.20 (18.35)	0.20 (1.00)	−1.86	0.056^*^	−1.36

Significant results in bold;

* statistical trend.

Next, analyses were conducted to see if tumor type differentiated patients in terms of cognitive functioning. It turned out that patients with oligodendrogliomas made more errors on the WCST test (*t* = 2.11; *p* = 0.03), but this only concerned perseverative errors (*t* = 1.92; *p* = 0. 04), gave fewer conceptual responses (*t* = −4.08; *p* = 0.002), completed fewer categories (*t* = −2.54; *p* = 0.01), and scored lower on the Learning to learn index (*t* = −1.98; *p* = 0.04).

Finally, we assessed whether the presence of epileptic seizures was relevant to neuropsychological outcomes. We found that patients with epileptic seizures made more errors on the WCST test (*t* = 2.45; *p* = 0.02), and it applied to both perseverative (*t* = 2.19; *p* = 0.03) and non-perseverative errors (*t* = 2.05; *p* = 0.04). They also had a lower number of completed categories (*t* = −2.54; *p* = 0.01).

## Discussion

The aim of the present study was to conduct an in-depth examination of the executive function profile of patients with tumors in the supplementary motor area. Due to the very limited number of studies on this topic to date, it was decided to compare the obtained results with a control group of healthy individuals, which, to the best of our knowledge, has not been conducted on such a scale before (previous studies had limitations in terms of a narrow scope of executive function assessment, small sample size, or lack of a control group). Four commonly used tests to assess various aspects of executive functions were employed for this purpose. It has been shown that significant intergroup differences occurred in each of the tests, but not in every indicator, showing a wide range of disorders, but also their high specificity.

In the Verbal Fluency Test, significant differences were demonstrated only in two out of three tasks; patients scored lower in the phonemic and switching criteria, while their results did not differ significantly in the semantic criterion. As previous studies have shown, these two tasks are more strongly saturated with the executive function factor, while semantic fluency is more significantly influenced by memory (Delgado-Álvarez et al., 2021). Reduced performance on the verbal fluency test was also observed in other studies concerning the functioning of patients with SMA tumors (Cañas et al., 2018; Sjöberg et al., 2019; Tymowski et al., 2018). Moreover, NIRS studies showed strong correlation between word retrieval and responses of posterior SMA (Obayashi, 2020). SMA complex connects with inferior frontal gyrus (IFG) via frontal aslant tract (FAT), and it is well known, that the verbal fluency task involves IFG and SMA, where SMA probably provides the executive control of word retrieval and IFG is involved in articulation and speech production (Alario et al., 2006; Catani et al., 2012, 2013; Costafreda et al., 2006; Obayashi, 2020). This finding was supported also by a research using positron emission tomography (PET), which showed activation of SMA after performing verbal fluency task (Ravnkilde et al., 2002).

The performance of the Stroop Test showed that the patients had similar to healthy subjects average time of completing each part of the test, but what differentiated both groups was the interference score, which is the ratio of the time taken to complete the Stroop task (color-word naming) to the control task (color dots naming). This index is the most sensitive measure of executive functions, particularly inhibitory control. This score measures the ability to suppress automatic responses (like reading the word) in favor of a more controlled response (naming the ink color), which is one of the key aspects of executive functions (Chafetz and Matthews, 2004; Scarpina and Tagini, 2017). Patients also made more errors in the Stroop task (third part of the test) than healthy individuals, which may be caused by insufficient cognitive control and reduced ability to inhibit automatic reactions. Functional studies have shown pre-SMA activation during tasks requiring participants to choose between competing responses or modify planned actions in response to novel information. As described in other studies, the pre-SMA plays a crucial role in action inhibition when determining whether to execute or withhold from an activity (Wolpe et al., 2022). It was previously shown in fMRI investigations of motor inhibition tasks, which necessitates action cancellation and the go/no-go test that requires action prevention (Rae et al., 2014; Wolpe et al., 2022). The effect was also present in studies describing effects of transcranial magnetic stimulation of the pre-SMA which led to the impairment of effective action cancellation (Floden and Stuss, 2006). Inhibition is one of the critical executive functions that allows individuals to suppress inappropriate behavior, adjust action and maintain focus on task-relevant goals. Lesions or disruptions in the pre-SMA are linked to increased impulsivity and a reduced ability to inhibit responses (Chen et al., 2010; Wolpe et al., 2022). Study performed by Ravnkilde et al. (2002) with the use of PET showed that Stroop Test activated among others supplementary motor cortex. The results of previous study on patients with SMA tumors showed that they gained lower scores in Stroop Test than healthy controls (Sjöberg et al., 2019), which is congruent with our findings.

In the Tower of London Test, patients in the clinical group obtained significantly fewer correct answers, that is tasks solved with the minimum number of moves, they also made a greater number of excessive moves and committed more errors, i.e., rule violations. The time taken to complete the task did not differ significantly between the groups, but a statistical trend was observed. To our knowledge, no study has yet been conducted using this test to examine patients with SMA tumors. Experimental studies, however, have shown activation of the SMA during the performance of TOL, which was observed in fMRI study, where task load was associated with the increased activity of this structure (Van den Heuvel et al., 2003) and PET, in which, interestingly, the levels of relative regional cerebral blood flow (rrCBF) correlated with the number of moves made during the scan (Dagher et al., 1999). The supplementary motor area and its connections to the right putamen make inhibitory control and planning for flexible, goal-oriented, adaptive behavior possible, allowing one to control one's movements and set goals (Baraniuk et al., 2024). The involvement of the SMA in performing the TOL may partly result from the motor nature of this task—what the subject has to do is plan and execute a sequence of movements, simultaneously monitoring the achieved effect and making any necessary adjustments (Dagher et al., 1999). Correct performance of this task depends on the efficient functioning of executive functions.

In the WCST, patients with SMA tumors scored worse than healthy individuals on numerous indicators, namely it took them longer (more trials) to complete the test, they had fewer correct answers and more perseveration, fewer completed categories, a higher rate of failure to maintain set, and a lower rate of learning from their mistakes and provided feedback. To our knowledge, only one study has been conducted so far involving a population of patients with SMA tumors who were assessed using the WCST (Tymowski et al., 2018), but the results obtained in that study are inconsistent with those obtained in the present study, suggesting the need for further research. The correct execution of the WSCT test requires participants to perform a series of evaluations based on feedback and make decisions based on the conducted analyses. Based on the previous studies, the hypothesis was put forward that the SMA may function as a bridge between the corticospinal tract and systems that facilitate executive function, which are generally regarded as instruments for making conscious decisions (Sjöberg, 2021). In turn, the study conducted by Wang et al. (2015) discovered SMA activation in response to negative feedback. Interestingly, this structure was not active during the performance of the WCST when negative feedback was absent. The authors postulate that the activation of the SMA presumably supported the process of searching for a new hypothesis, developing a new response scheme to the stimulus and facilitating a rule-based response. Similar conclusions regarding SMA activation come from the study by Nagahama et al. (1996), who detected an increase in rCBF in healthy subjects performing the WCST.

The analysis of the relationship between tumor size and neuropsychological test results showed a significant correlation in the case of TOL and WCST, and no correlation with the results of the Stroop test and the Verbal Fluency Test. There are some studies which have shown a link between tumor size and the level of cognitive functioning (Gempt et al., 2017; Noll et al., 2016; van Kessel et al., 2019). The discrepancy in the results obtained in this study may be caused by the varying complexity of the individual tasks. The Stroop Test and the Verbal Fluency Test are relatively easier to perform and involve fewer functions than the Tower of London Test and the Wisconsin Card Sorting Test. Correct execution of the Stroop Test mainly requires efficient inhibition of automatic, impulsive reactions, while the VFT requires efficient, systematic searching of semantic memory stores and switching between categories. Meanwhile, both TOL and WCST, in addition to inhibition and switching, also involve, among other things, reasoning, planning, and cognitive flexibility. Perhaps for this reason, this structure is more heavily burdened, and greater damage causes a stronger overload of the entire system and a decline in the level of function performance, while in the case of simpler tasks, it is possible to compensate for the deficits for a longer time, hence the presence of intergroup differences but the lack of evident correlations with tumor size.

A comparison of neuropsychological performance by individuals with tumors in the left or right hemisphere showed that significant differences occurred only in selected WCST indicators, where individuals with tumors in the left hemisphere scored significantly lower than those with tumors in the right hemisphere. The observed lateralization pattern suggests greater involvement of the left SMA in executive functions. The left SMA has been proven to be associated with language (Hertrich et al., 2016). Verbal skills, in turn, can moderate performance in internally guided action selection, cognitive flexibility, language-related reasoning, and working memory (Baldo et al., 2010; du Boisgueheneuc et al., 2006). These factors could influence complex problem-solving tasks, such as the WCST. Previous studies provide conflicting data regarding the neuronal lateralization associated with performing WCST. Kawasaki et al. (1993) detected increased blood flow associated with this test in the left prefrontal area, Marenco et al. (1993) and Volz et al. (1997) in the right prefrontal area, and Sumitani et al. (2006) mainly bilaterally. Clear conclusions are lacking, and the functional asymmetry of the SMA remains under-explored in clinical populations. Further studies are required to clarify the specific contributions of each hemisphere to executive functioning, especially in patients with lesions.

Patients with oligodendrogliomas performed significantly worse on certain neuropsychological tests than those with astrocytomas. The infiltrative nature of oligodendrogliomas, along with their direct effect on neuronal integrity, may result in more extensive disruption of subcortical tracts than astrocytomas, which tend to displace these pathways rather than infiltrate them (Landers et al., 2023). This infiltration could lead to greater cognitive deficits due to the disintegration of critical neural networks.

Our findings indicate that patients who experienced epileptic seizures performed worse on the WCST. This suggests that seizure activity may exacerbate executive dysfunction in this population. Previous studies have demonstrated that epilepsy and the use of antiepileptic drugs (AEDs) can negatively impact cognitive functions, including executive processes, in glioma patients (Klein et al., 2003). The presence of seizures may contribute to additional neural disruption beyond the tumor's effects, leading to more pronounced cognitive deficits. It should be emphasized that differences in test performance were noted only in the WCST. In other tests, patients performed similarly to those without seizures. This may be because most patients diagnosed with epilepsy had seizures for a limited time before receiving a brain tumor diagnosis and undergoing surgery. Perhaps the brief exposure to the damaging effects of epilepsy caused only some neuronal networks to deteriorate. However, given the size of the group, this issue requires further research.

Although many of the analyses achieved a level of statistical significance, some of the results showed only a statistical trend. We decided to leave those results in the article as information of possible clinical significance, which should be considered not as a final conclusion but as an indication that the issue requires further investigation in future studies.

It is important to acknowledge the limitations of the study. They include a relatively small sample size of participants especially when considering the heterogeneity of tumor locations (left vs. right SMA) and the variability in tumor volume. This limits the generalizability of the findings. Increasing the number of participants would make it possible to conduct advanced statistical analyses that could shed a new light on the results and increase the precision of the conclusions. Moreover, to improve the present study, it would be beneficial to obtain socioeconomic data, such as income or place of residence, which could enrich our clinical observations with social context. Including data from tractography and functional neuroimaging would enhance the accuracy of our findings and the conclusions, as well as adding analyses based on molecular data of gliomas and their relationship to cognitive functioning status.

## Conclusions

The supplementary motor area, which by its very name may be mistakenly associated solely with movement, is a structure with a very rich network of functional connections, and in the case of damage, it has an abundant and diverse symptomatology that significantly extends beyond the motor sphere. The conducted study showed that patients with tumors in this area present a wide range of executive dysfunctions, including planning, reasoning, inhibition, switching, and cognitive flexibility. There are still very few studies exploring the spectrum and depth of disorders observed in patients with SMA damage, but this research direction seems very promising. In the future, it is crucial to gather a larger group of patients and conduct comprehensive studies that, in addition to neuropsychological examinations, also include advanced neuroimaging analyses, intraoperative mapping, and follow-up. From a clinical perspective, the presence of executive dysfunctions in patients with SMA tumors highlights the need for routine neuropsychological monitoring before and after surgery. Identifying difficulties in cognitive flexibility, planning, or response inhibition early on can guide postoperative care and rehabilitation, support planning for a return to work or independent functioning, as well as inform caregiver education. Continuation and development of this research with larger cohorts would provide an opportunity to expand knowledge about the role of this brain structure, and from a practical standpoint, it would facilitate neurosurgeons in planning surgeries, give neuropsychologists diagnostic guidelines, thereby giving patients a chance to protect cognitive functions and improve their quality of life.

## Data Availability

The raw data supporting the conclusions of this article will be made available by the authors, without undue reservation.

## References

[B1] Alario F. X. Chainay H. Lehericy S. Cohen L. (2006). The role of the supplementary motor area (SMA) in word production. Brain Res. 1076, 129–143. 10.1016/j.brainres.2005.11.104 16480694

[B2] Aron A. R. Behrens T. E. Smith S. Frank M. J. Poldrack R. A. (2007). Triangulating a cognitive control network using diffusion-weighted magnetic resonance imaging (MRI) and functional MRI. J. Neurosci. 27, 3743–3752. 10.1523/JNEUROSCI.0519-07.2007 17409238 PMC6672420

[B3] Bala A. Olejnik A. Gottman-Narożna A. Rejner W. Koczyk K. Dziedzic T.. (2025). Deficits of attention and working memory in patients with gliomas of supplementary motor area. J. Clin. Med. 14:1229. 10.3390/jcm14041229 40004759 PMC11856663

[B4] Baldo J. V. Bunge S. A. Wilson S. M. Dronkers N. F. (2010). Is relational reasoning dependent on language? A voxel-based lesion symptom mapping study. Brain Lang. 113, 59–64. 10.1016/j.bandl.2010.01.004 20206985 PMC2994599

[B5] Baraniuk J. N. Thapaliya K. Inderyas M. Shan Z. Y. Barnden L. R. (2024). Stroop task and practice effects demonstrate cognitive dysfunction in long COVID and myalgic encephalomyelitis/chronic fatigue syndrome. Sci. Rep. 14:26796. 10.1038/s41598-024-75651-3 39500939 PMC11538523

[B6] Bauchet L. Rigau V. Mathon B. Darlix A. With the participation of the Société Française de Neurochirurgie (SFNC), Club de Neuro-oncologie of SFNC, Société Française de Neuropathologie (SFNP) and Association des Neuro-Oncologues d'Expression Française (ANOCEF) (2024). Epidemiological analysis of adult-type diffuse lower-grade gliomas and incidence and prevalence estimates of diffuse IDH-mutant gliomas in France. Neuro-Chirurgie 71:101627. 10.1016/j.neuchi.2024.101627 39710298

[B7] Bellebaum C. Daum I. (2007). Cerebellar involvement in executive control. Cerebellum 6, 184–192. 10.1080/14734220601169707 17786814

[B8] Cañas A. Juncadella M. Lau R. Gabarrós A. Hernández M. (2018). Working memory deficits after lesions involving the supplementary motor area. Front. Psychol. 9:765. 10.3389/fpsyg.2018.00765 29875717 PMC5974158

[B9] Catani M. Dell'acqua F. Vergani F. Malik F. Hodge H. Roy P.. (2012). Short frontal lobe connections of the human brain. Cortex 48, 273–291. 10.1016/j.cortex.2011.12.001 22209688

[B10] Catani M. Mesulam M. M. Jakobsen E. Malik F. Martersteck A. Wieneke C.. (2013). A novel frontal pathway underlies verbal fluency in primary progressive aphasia. Brain 136, 2619–2628. 10.1093/brain/awt163 23820597 PMC3722349

[B11] Chafetz M. D. Matthews L. H. (2004). A new interference score for the Stroop test. Arch. Clin. Neuropsychol. 19, 555–567. 10.1016/j.acn.2003.08.004 15163456

[B12] Chen X. Scangos K. W. Stuphorn V. (2010). Supplementary motor area exerts proactive and reactive control of arm movements. J. Neurosci. 30, 14657–14675. 10.1523/JNEUROSCI.2669-10.2010 21048123 PMC2990193

[B13] Cona G. Semenza C. (2017). Supplementary motor area as key structure for domain-general sequence processing: a unified account. Neurosci. Biobehav. Rev. 72, 28–42. 10.1016/j.neubiorev.2016.10.033 27856331

[B14] Costafreda S. G. Fu C. H. Lee L. Everitt B. Brammer M. J. David A. S.. (2006). A systematic review and quantitative appraisal of fMRI studies of verbal fluency: role of the left inferior frontal gyrus. Hum. Brain Mapp. 27, 799–810. 10.1002/hbm.20221 16511886 PMC6871344

[B15] Coull J. T. Vidal F. Burle B. (2016). When to act, or not to act: that's the SMA's question. Curr. Opin. Behav. Sci. 8, 14–21. 10.1016/j.cobeha.2016.01.003

[B16] Courson M. Macoir J. Tremblay P. (2017). Role of medial premotor areas in action language processing in relation to motor skills. Cortex 95, 77–91. 10.1016/j.cortex.2017.08.002 28858609

[B17] Cristofori I. Cohen-Zimerman S. Grafman J. (2019). “Executive functions,” in Handbook of Clinical Neurology, Vol. 163, ed. Grafman J. (Amsterdam: Elsevier), 197–219. 10.1016/B978-0-12-804281-6.00011-2 31590731

[B18] Dagher A. Owen A. M. Boecker H. Brooks D. J. (1999). Mapping the network for planning: a correlational PET activation study with the Tower of London ask. Brain J. Neurol. 122, 1973–1987. 10.1093/brain/122.10.1973 10506098

[B19] Delgado-Álvarez A. Matias-Guiu J. A. Delgado-Alonso C. Hernández-Lorenzo L. Cortés-Martínez A. Vidorreta L.. (2021). Cognitive processes underlying verbal fluency in multiple sclerosis. Front. Neurol. 11:629183. 10.3389/fneur.2020.629183 33551984 PMC7859643

[B20] Dominik T. Mele A. Schurger A. Maoz U. (2024). Libet's legacy: a primer to the neuroscience of volition. Neurosci. Biobehav. Rev. 157:105503. 10.1016/j.neubiorev.2023.105503 38072144

[B21] du Boisgueheneuc F. Levy R. Volle E. Seassau M. Duffau H. Kinkingnehun S.. (2006). Functions of the left superior frontal gyrus in humans: a lesion study. Brain J. Neurol. 129, 3315–3328. 10.1093/brain/awl244 16984899

[B22] Duann J. R. Ide J. S. Luo X. Li C. S. (2009). Functional connectivity delineates distinct roles of the inferior frontal cortex and presupplementary motor area in stop signal inhibition. J. Neurosci. 29, 10171–10179. 10.1523/JNEUROSCI.1300-09.2009 19675251 PMC2769086

[B23] Duffau H. Capelle L. (2004). Preferential brain locations of low-grade gliomas. Cancer 100, 2622–2626. 10.1002/cncr.20297 15197805

[B24] Ek L. Elwin M. Neander K. (2024). Neuropsychological longitudinal study of patients with low-grade gliomas: cognitive impairment. Appl. Neuropsychol. Adult 1–11. 10.1080/23279095.2024.2325546. [Epub ahead of print]. 38470840

[B25] Fedota J. R. Hardee J. E. Pérez-Edgar K. Thompson J. C. (2014). Representation of response alternatives in human presupplementary motor area: multi-voxel pattern analysis in a go/no-go task. Neuropsychologia 56, 110–118. 10.1016/j.neuropsychologia.2013.12.022 24440411 PMC3966967

[B26] Floden D. Stuss D. T. (2006). Inhibitory control is slowed in patients with right superior medial frontal damage. J. Cogn. Neurosci. 18, 1843–1849. 10.1162/jocn.2006.18.11.1843 17069475

[B27] Florio T. M. Scarnati E. Rosa I. Di Censo D. Ranieri B. Cimini A.. (2018). The basal ganglia: more than just a switching device. CNS Neurosci. Therapeut. 24, 677–684. 10.1111/cns.12987 29879292 PMC6490066

[B28] Forst D. A. Nahed B. V. Loeffler J. S. Batchelor T. T. (2014). Low-grade gliomas. Oncologist 19, 403–413. 10.1634/theoncologist.2013-0345 24664484 PMC3983820

[B29] Gempt J. Lange N. Bette S. Foreman S. C. Cammardella J. H. Albertshauser J.. (2017). Factors influencing neurocognitive function in patients with neuroepithelial tumors. Sci. Rep. 7:17764. 10.1038/s41598-017-17833-w 29259230 PMC5736700

[B30] Guida P. Foffani G. Obeso I. (2023). The supplementary motor area and automatic cognitive control: lack of evidence from two neuromodulation techniques. J. Cogn. Neurosci. 35, 439–451. 10.1162/jocn_a_01954 36603037

[B31] Heaton R. K. Chelune G. J. Talley J. L. Kay G. G. Curtis G. (1993). Wisconsin Card Sorting Test Manual: Revised and Expanded. Odessa, TX: Psychological Assessment Resources.

[B32] Heitzer A. M. Ashford J. M. Hastings C. Liu A. P. Y. Wu S. Bass J. K.. (2019). Neuropsychological outcomes of patients with low-grade glioma diagnosed during the first year of life. J. Neurooncol. 141, 413–420. 10.1007/s11060-018-03048-0 30467811 PMC6344293

[B33] Hertrich I. Dietrich S. Ackermann H. (2016). The role of the supplementary motor area for speech and language processing. Neurosci. Biobehav. Rev. 68, 602–610. 10.1016/j.neubiorev.2016.06.030 27343998

[B34] Kawasaki Y. Maeda Y. Suzuki M. Urata K. Higashima M. Kiba K.. (1993). SPECT analysis of regional cerebral blood flow changes in patients with schizophrenia during the Wisconsin Card Sorting Test. Schizophr. Res. 10, 109–116. 10.1016/0920-9964(93)90045-K 8398942

[B35] Klein M. Engelberts N. H. van der Ploeg H. M. Kasteleijn-Nolst Trenité D. G. Aaronson N. K. Taphoorn M. J.. (2003). Epilepsy in low-grade gliomas: the impact on cognitive function and quality of life. Ann. Neurol. 54, 514–520. 10.1002/ana.10712 14520665

[B36] Landers M. J. F. Brouwers H. B. Kortman G. J. Boukrab I. Baene D. e. Rutten W.. (2023). Oligodendrogliomas tend to infiltrate the frontal aslant tract, whereas astrocytomas tend to displace it. Neuroradiology 65, 1127–1131. 10.1007/s00234-023-03153-6 37127719 PMC10271893

[B37] Lanese A. Franceschi E. Brandes A. A. (2018). The risk assessment in low-grade gliomas: an analysis of the European Organization for Research and Treatment of Cancer (EORTC) and the Radiation Therapy Oncology Group (RTOG) criteria. Oncol. Ther. 6, 105–108. 10.1007/s40487-018-0063-9 32700027 PMC7359968

[B38] Lezak M. D. (2004). Neuropsychological Assessment. Oxford: Oxford University Press.

[B39] Lezak M. D. Howieson D. B. Bigler E. D. Tranel D. (2012). Neuropsychological Assessment (5th ed.). Oxford: Oxford University Press.

[B40] Logue S. F. Gould T. J. (2014). The neural and genetic basis of executive function: attention, cognitive flexibility, and response inhibition. Pharmacol. Biochem. Behav. 123, 45–54. 10.1016/j.pbb.2013.08.007 23978501 PMC3933483

[B41] Lu M. T. Preston J. B. Strick P. L. (1994). Interconnections between the prefrontal cortex and the premotor areas in the frontal lobe. J. Compar. Neurol. 341, 375–392. 10.1002/cne.903410308 7515081

[B42] Makoshi Z. Kroliczak G. van Donkelaar P. (2011). Human supplementary motor area contribution to predictive motor planning. J. Motor Behav. 43, 303–309. 10.1080/00222895.2011.584085 21732868

[B43] Marenco S. Coppola R. Daniel D. G. Zigun J. R. Weinberger D. R. (1993). Regional cerebral blood flow during the Wisconsin Card Sorting Test in normal subjects studied by xenon-133 dynamic SPECT: comparison of absolute values, percent distribution values, and covariance analysis. Psychiatry Res. 50, 177–192. 10.1016/0925-4927(93)90029-H 8272453

[B44] Nachev P. Kennard C. Husain M. (2008). Functional role of the supplementary and pre-supplementary motor areas. Nat. Rev. Neurosci. 9, 856–869. 10.1038/nrn2478 18843271

[B45] Nagahama Y. Fukuyama H. Yamauchi H. Matsuzaki S. Konishi J. Shibasaki H.. (1996). Cerebral activation during performance of a card sorting test. Brain J. Neurol. 119, 1667–1675. 10.1093/brain/119.5.1667 8931588

[B46] Noll K. R. Ziu M. Weinberg J. S. Wefel J. S. (2016). Neurocognitive functioning in patients with glioma of the left and right temporal lobes. J. Neuro Oncol. 128, 323–331. 10.1007/s11060-016-2114-0 27022915 PMC4884162

[B47] Obayashi S. (2020). The supplementary motor area responsible for word retrieval decline after acute thalamic stroke revealed by coupled SPECT and near-infrared spectroscopy. Brain Sci. 10:247. 10.3390/brainsci10040247 32331319 PMC7226437

[B48] Oda H. Sawaguchi Y. Kunimura H. Kawasaki T. Hiraoka K. (2021). Supplementary motor area contributes to carrying previous movement information over to current movement. Neuroreport 32, 223–227. 10.1097/WNR.0000000000001578 33395190

[B49] Olejnik A. Bala A. Dziedzic T. Rysz A. Marchel A. Kunert P. (2024). Executive dysfunction profile in mesial temporal lobe epilepsy. Acta Neuropsychol. 22, 1–13. 10.5604/01.3001.0053.9737

[B50] Ortuño F. M. López P. Ojeda N. Cervera S. (2005). Dysfunctional supplementary motor area implication during attention and time estimation tasks in schizophrenia: a PET-O15 water study. NeuroImage 24, 575–579. 10.1016/j.neuroimage.2004.09.010 15627600

[B51] Palmisciano P. Haider A. S. Balasubramanian K. Dadario N. B. Robertson F. C. Silverstein J. W.. (2022). Supplementary motor area syndrome after brain tumor surgery: a systematic review. World Neurosurg. 165, 160–171.e2. 10.1016/j.wneu.2022.06.080 35752423

[B52] Piervincenzi C. Suppa A. Petsas N. Fabbrini A. Trebbastoni A. Asci F.. (2023). Parkinsonism is associated with altered SMA-basal ganglia structural and functional connectivity in frontotemporal degeneration. Biomedicines 11:522. 10.3390/biomedicines11020522 36831058 PMC9953061

[B53] Rae C. L. Hughes L. E. Weaver C. Anderson M. C. Rowe J. B. (2014). Selection and stopping in voluntary action: ameta-analysis and combined fMRI study. NeuroImage 86, 381–391. 10.1016/j.neuroimage.2013.10.012 24128740 PMC3898966

[B54] Ravnkilde B. Videbech P. Rosenberg R. Gjedde A. Gade A. (2002). Putative tests of frontal lobe function: a PET-study of brain activation during Stroop's Test and verbal fluency. J. Clin. Exp. Neuropsychol., 24, 534–547. 10.1076/jcen.24.4.534.1033 12187466

[B55] Rimmer B. Balla M. Dutton L. Williams S. Lewis J. Gallagher P.. (2024). “It changes everything”: understanding how people experience the impact of living with a lower-grade glioma. Neuro-Oncol. Pract. 11, 255–265. 10.1093/nop/npae006 38737616 PMC11085834

[B56] Rushworth M. F. Hadland K. A. Paus T. Sipila P. K. (2002). Role of the human medial frontal cortex in task switching: a combined fMRI and TMS study. J. Neurophysiol. 87, 2577–92. 10.1152/jn.2002.87.5.2577 11976394

[B57] Scarpina F. Tagini S. (2017). The Stroop Color and Word Test. Front. Psychol. 8:557. 10.3389/fpsyg.2017.00557 28446889 PMC5388755

[B58] Shallice T. (1982). Specific impairments of planning. Philos. Transac. Royal Soc. London Ser. B Biol. Sci. 298, 199–209. 10.1098/rstb.1982.0082 6125971

[B59] Sjöberg R. L. (2021). Free will and neurosurgical resections of the supplementary motor area: a critical review. Acta Neurochir. 163, 1229–1237. 10.1007/s00701-021-04748-9 33566193 PMC8053652

[B60] Sjöberg R. L. Stålnacke M. Andersson M. Eriksson J. (2019). The supplementary motor area syndrome and cognitive control. Neuropsychologia 129, 141–145. 10.1016/j.neuropsychologia.2019.03.013 30930302

[B61] Spreen O. Strauss E. (1998). A Compendium of Neuropsychological Tests (2nd ed.). New York: Oxford University Press.

[B62] Stroop J. R. (1935). Studies of interference in serial verbal reactions. J. Exp. Psychol. 18, 643–662. 10.1037/h0054651

[B63] Stuss D. T. (2011). Functions of the frontal lobes: relation to executive functions. J. Int. Neuropsychol. Soc. 17, 759–765. 10.1017/S1355617711000695 21729406

[B64] Sumitani S. Tanaka T. Tayoshi S. Ota K. Kameoka N. Ueno S.. (2006). Activation of the prefrontal cortex during the Wisconsin Card Sorting Test as measured by multichannel near-infrared spectroscopy. Neuropsychobiology 53, 70–76. 10.1159/000091722 16511337

[B65] Takeuchi H. Taki Y. Sassa Y. Hashizume H. Sekiguchi A. Fukushima A.. (2013). Brain structures associated with executive functions during everyday events in a non-clinical sample. Brain Struct. Funct. 218, 1017–1032. 10.1007/s00429-012-0444-z 22851058 PMC3695328

[B66] Tymowski M. Kaspera W. Metta-Pieszka J. Zarudzki Ł. Ładziński P. (2018). Neuropsychological assessment of patients undergoing surgery due to low-grade glioma involving the supplementary motor area. Clin. Neurol. Neurosurg. 175, 1–8. 10.1016/j.clineuro.2018.09.036 30292977

[B67] Van den Heuvel O. A. Groenewegen H. J. Barkhof F. Lazeron R. H. van Dyck R. Veltman D. J.. (2003). Frontostriatal system in planning complexity: a parametric functional magnetic resonance version of Tower of London task. NeuroImage 18, 367–374. 10.1016/S1053-8119(02)00010-1 12595190

[B68] Van der Werf Y. D. Scheltens P. Lindeboom J. Witter M. P. Uylings H. B. Jolles J.. (2003). Deficits of memory, executive functioning, and attention following infarction in the thalamus: a study of 22 cases with localized lesions. Neuropsychologia 41, 1330–1344. 10.1016/S0028-3932(03)00059-9 12757906

[B69] van Kessel E. Emons M. A. C. Wajer I. H. van Baarsen K. M. Broekman M. L. Robe P. A.. (2019). Tumor-related neurocognitive dysfunction in patients with diffuse glioma: a retrospective cohort study prior to antitumor treatment. Neuro Oncol. Pract. 6, 463–472. 10.1093/nop/npz008 31832216 PMC6899056

[B70] Volz H. P. Gaser C. Häger F. Rzanny R. Mentzel H. J. Kreitschmann-Andermahr I.. (1997). Brain activation during cognitive stimulation with the Wisconsin Card Sorting Test–a functional MRI study on healthy volunteers and schizophrenics. Psychiatry Res. 75, 145–157. 10.1016/S0925-4927(97)00053-X 9437772

[B71] Wang J. Cao B. Cai X. Gao H. Li F. (2015). Brain activation of negative feedback in rule acquisition revealed in a segmented wisconsin card sorting test. PLoS ONE 10:e0140731. 10.1371/journal.pone.0140731 26469519 PMC4607368

[B72] Welniarz Q. Gallea C. Lamy J. C. Méneret A. Popa T. Valabregue R.. (2019). The supplementary motor area modulates interhemispheric interactions during movement preparation. Hum. Brain Mapp. 40, 2125–2142. 10.1002/hbm.24512 30653778 PMC6865634

[B73] Wolpe N. Hezemans F. H. Rae C. L. Zhang J. Rowe J. B. (2022). The pre-supplementary motor area achieves inhibitory control by modulating response thresholds. Cortex 152, 98–108. 10.1016/j.cortex.2022.03.018 35550936

[B74] Xu C. Peng Y. Zhu W. Chen Z. Li J. Tan W.. (2022). An automated approach for predicting glioma grade and survival of LGG patients using CNN and radiomics. Front. Oncol. 12:969907. 10.3389/fonc.2022.969907 36033433 PMC9413530

